# Supplementary figures and images for: A spatio-temporal brain miRNA expression atlas identifies sex-independent age-related microglial driven miR-155-5p increase
Source: bioRxiv. 2025 Mar 16:2025.03.15.643430. Preprint. [Version 1] doi: 10.1101/2025.03.15.643430 (PMC11952541; doi:10.1101/2025.03.15.643430)

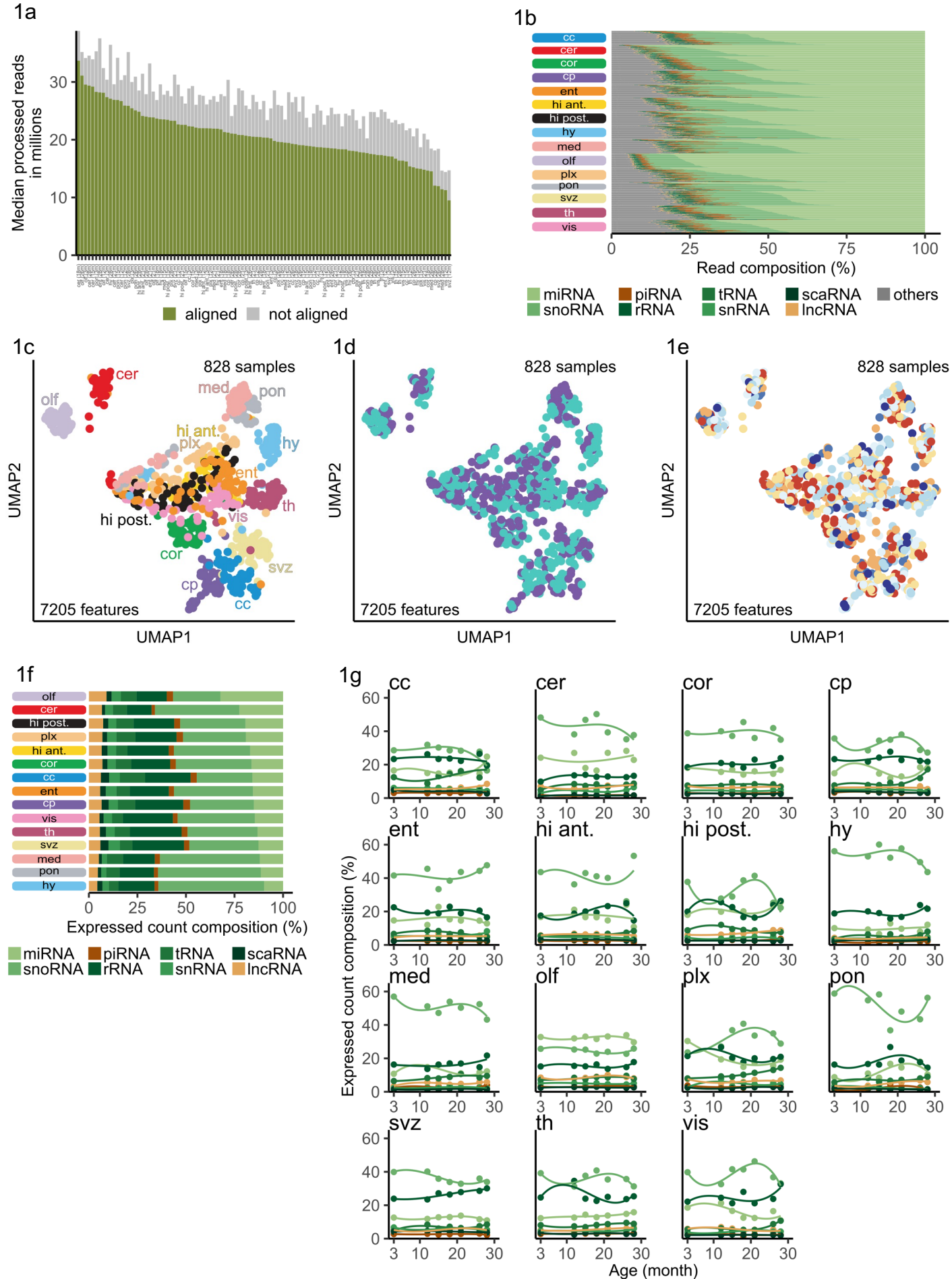

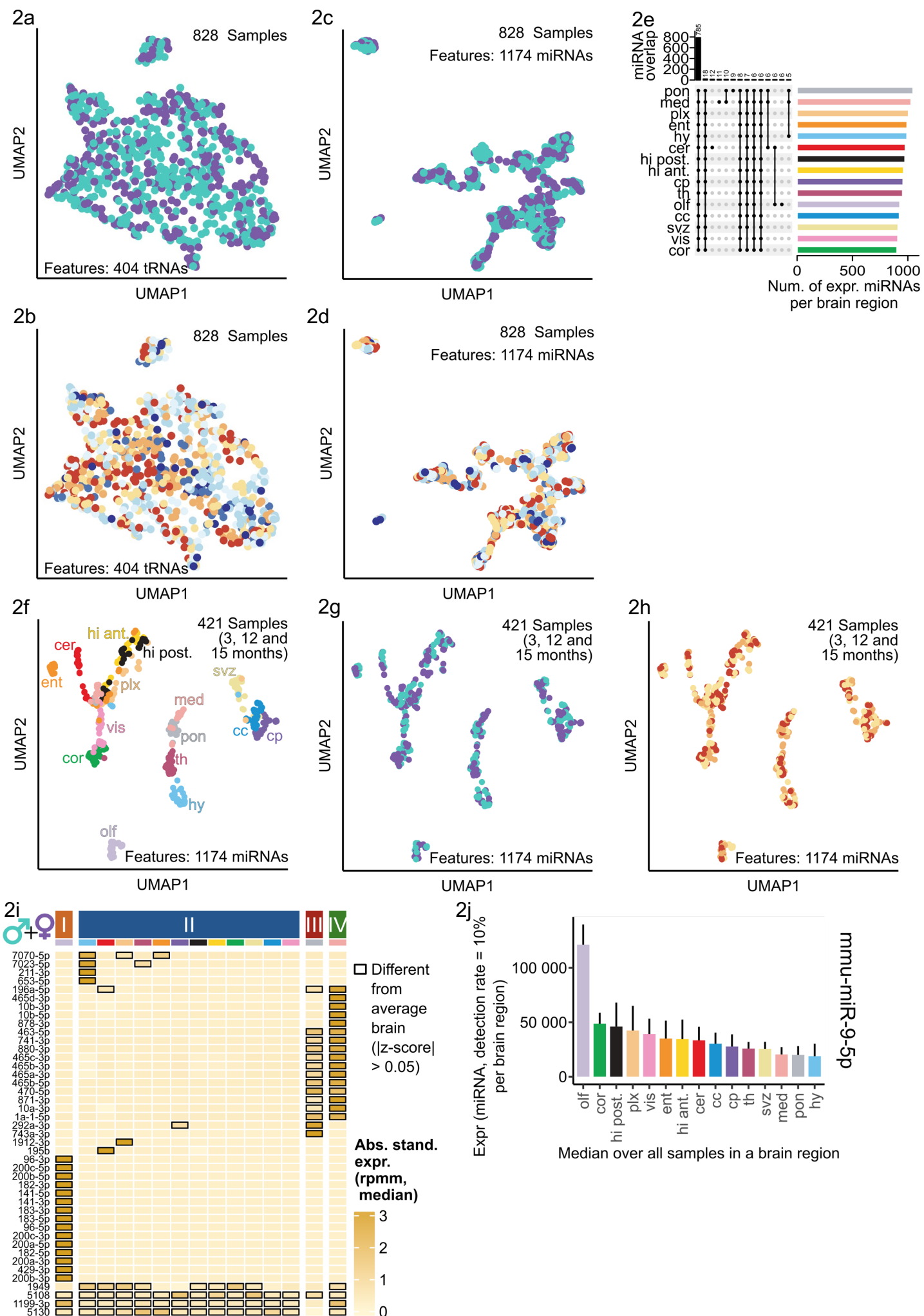

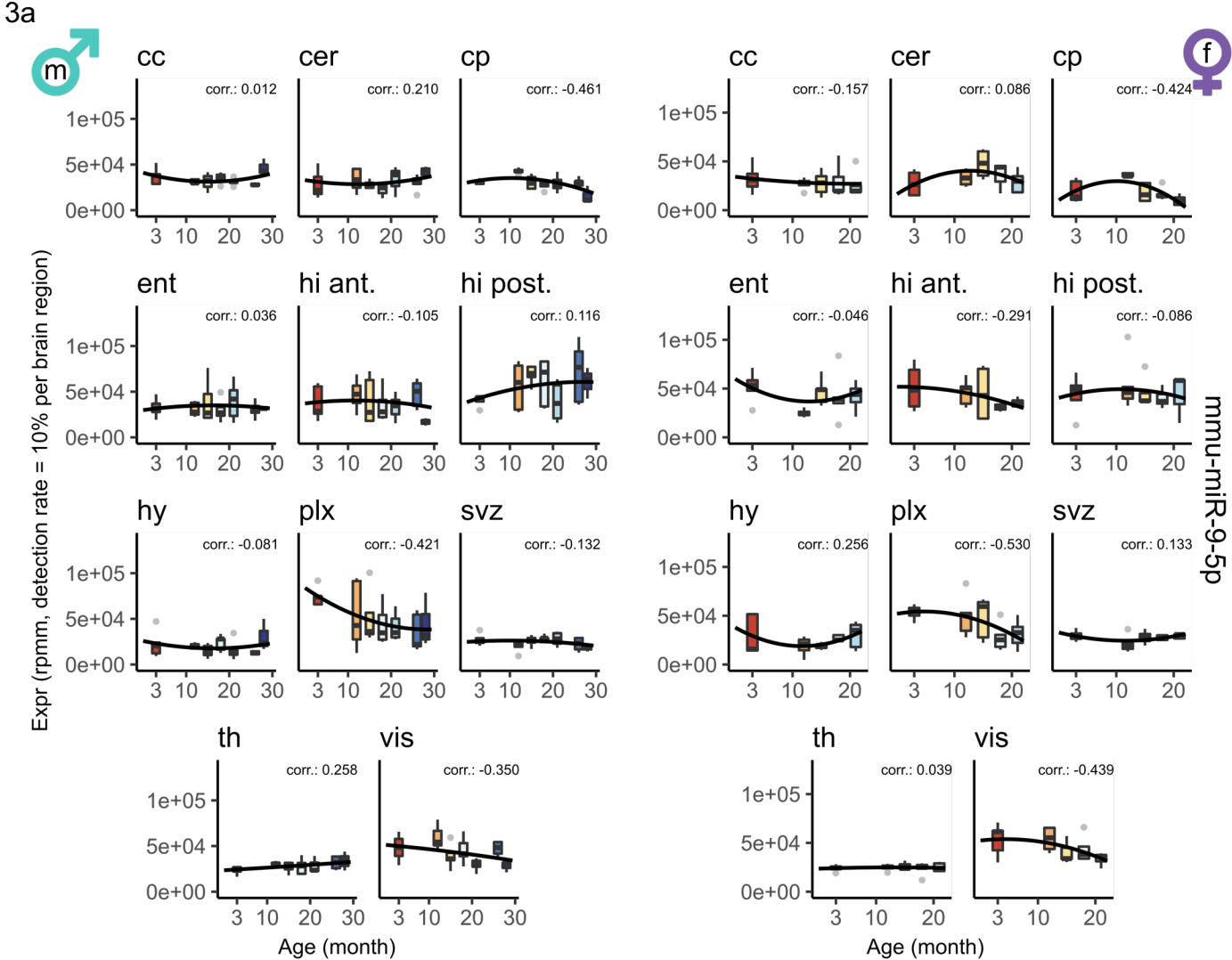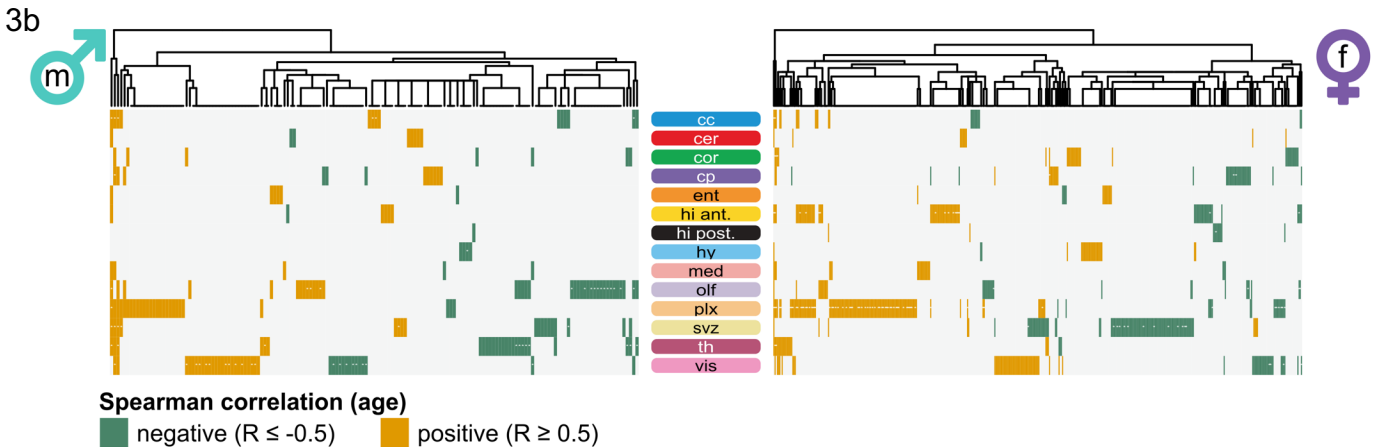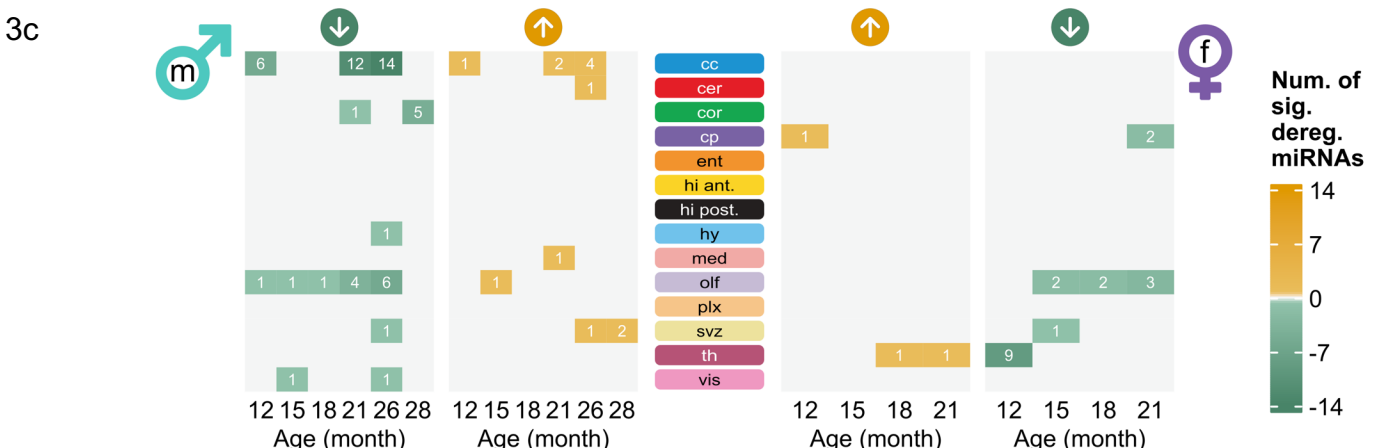

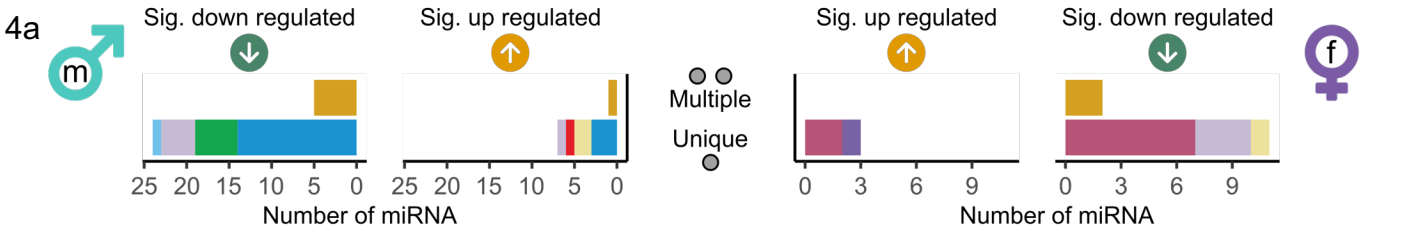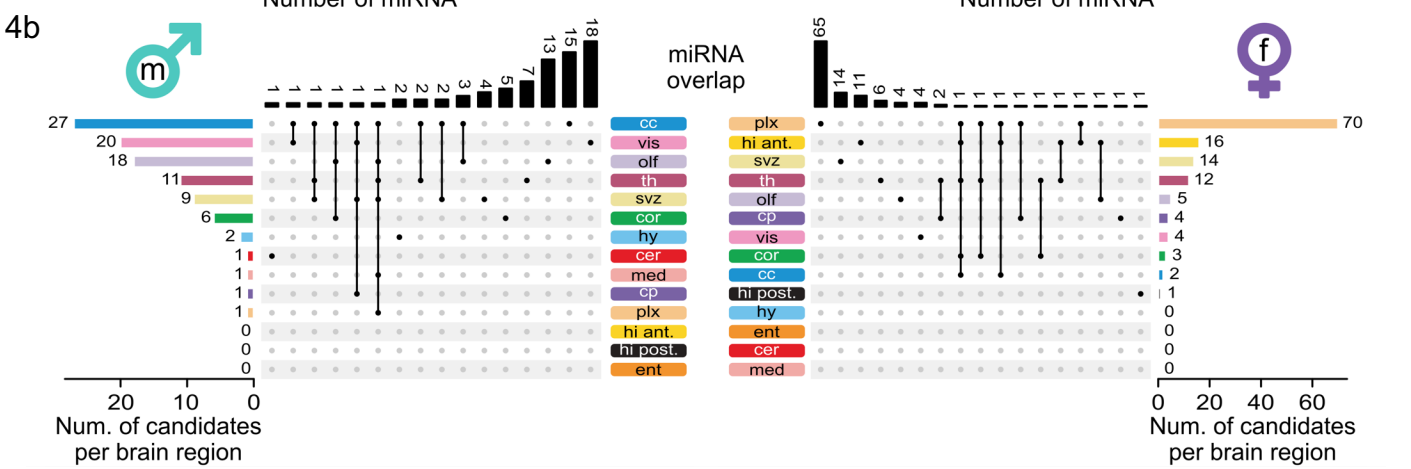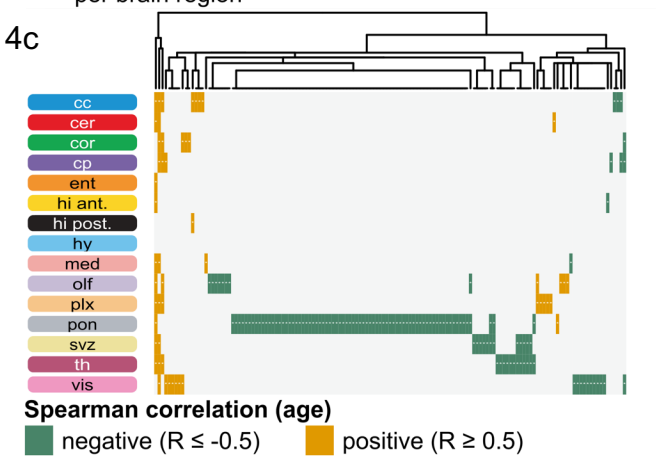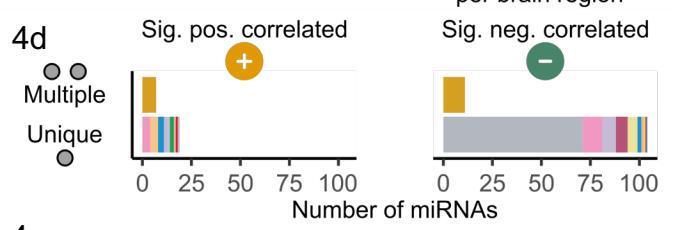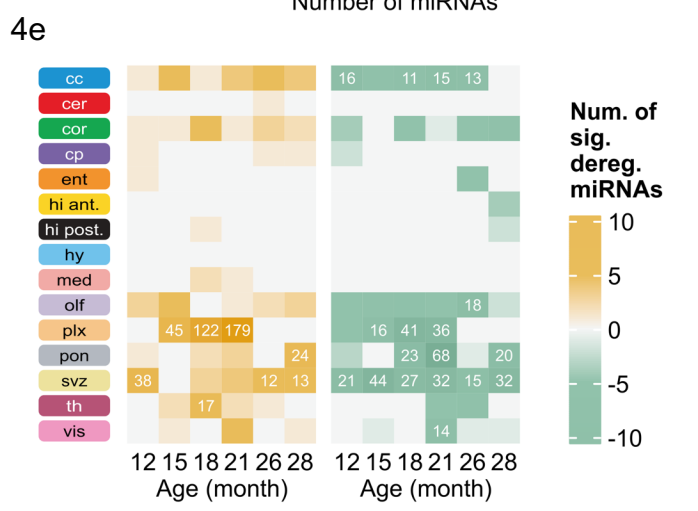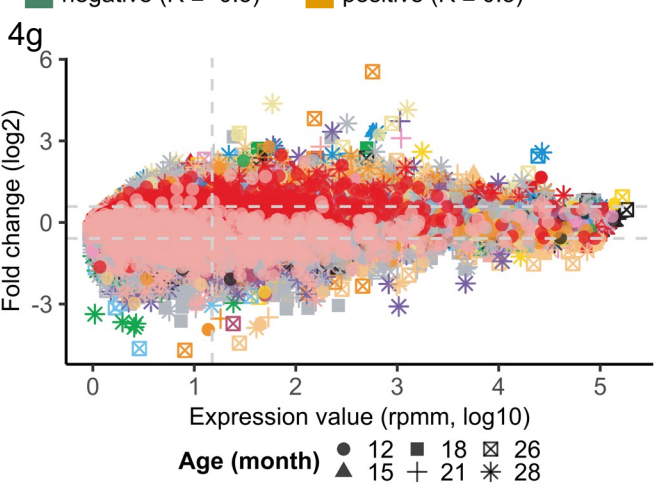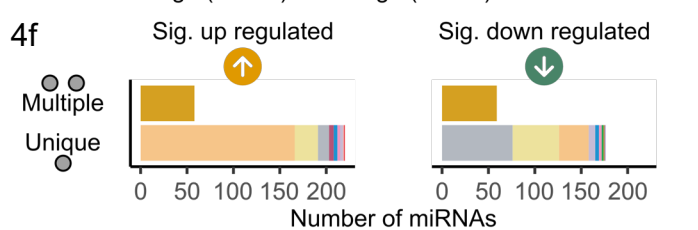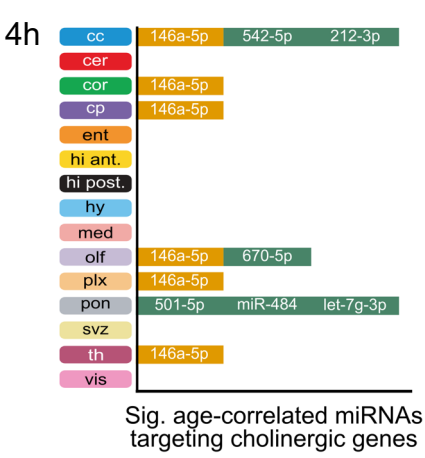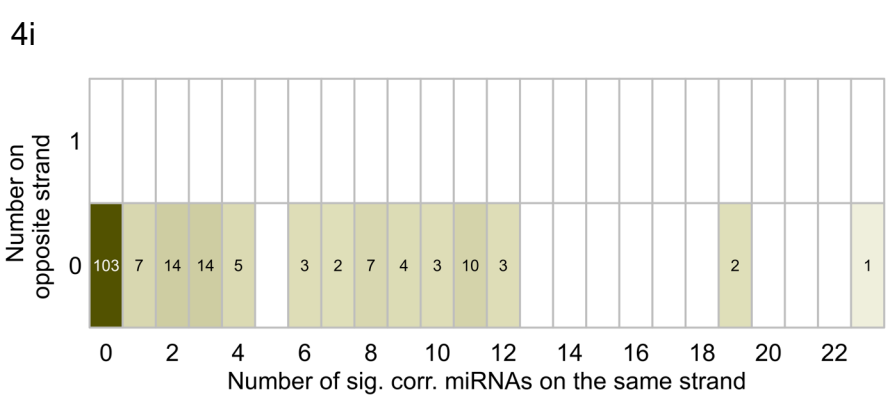

5a

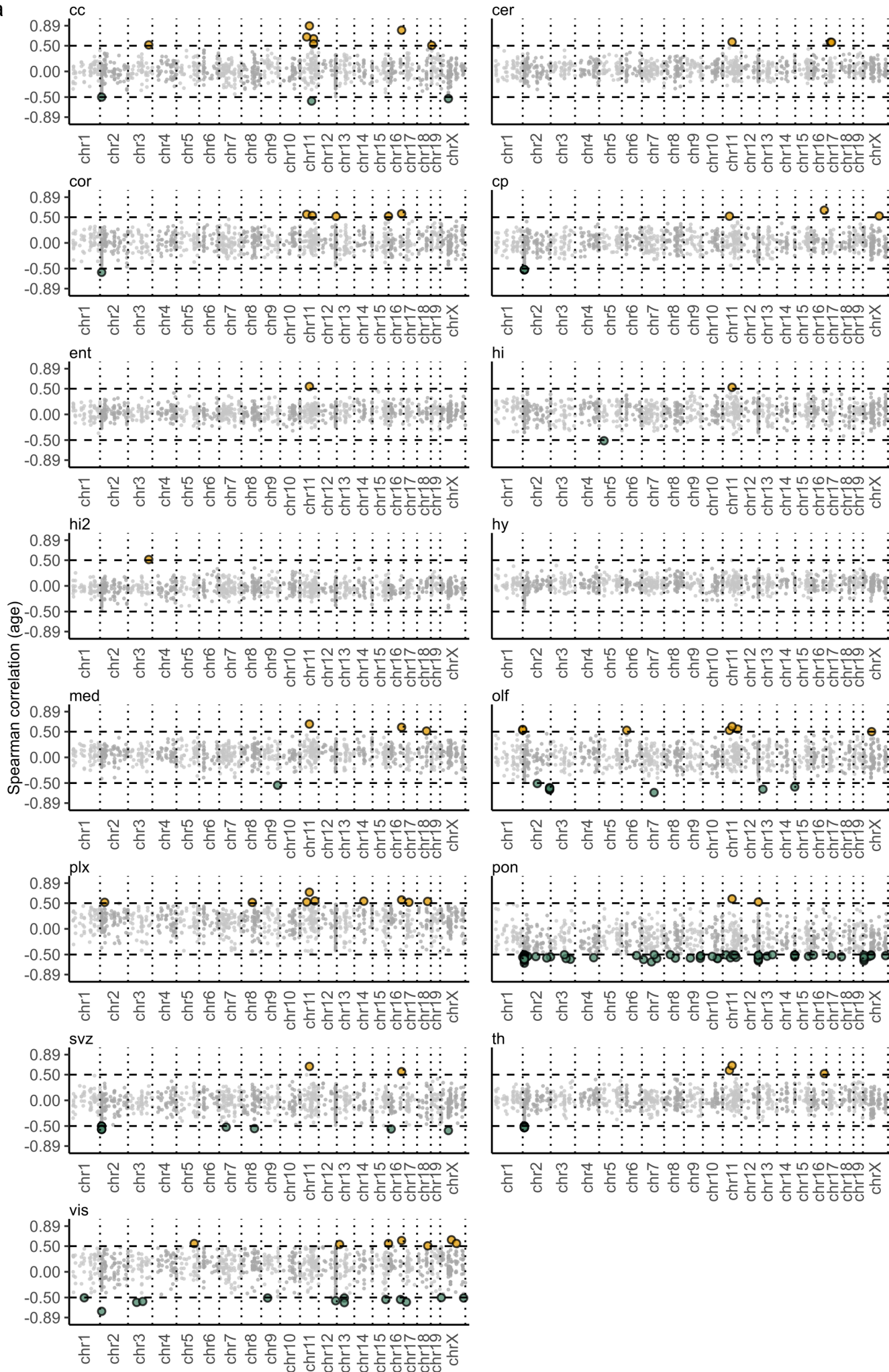

6a

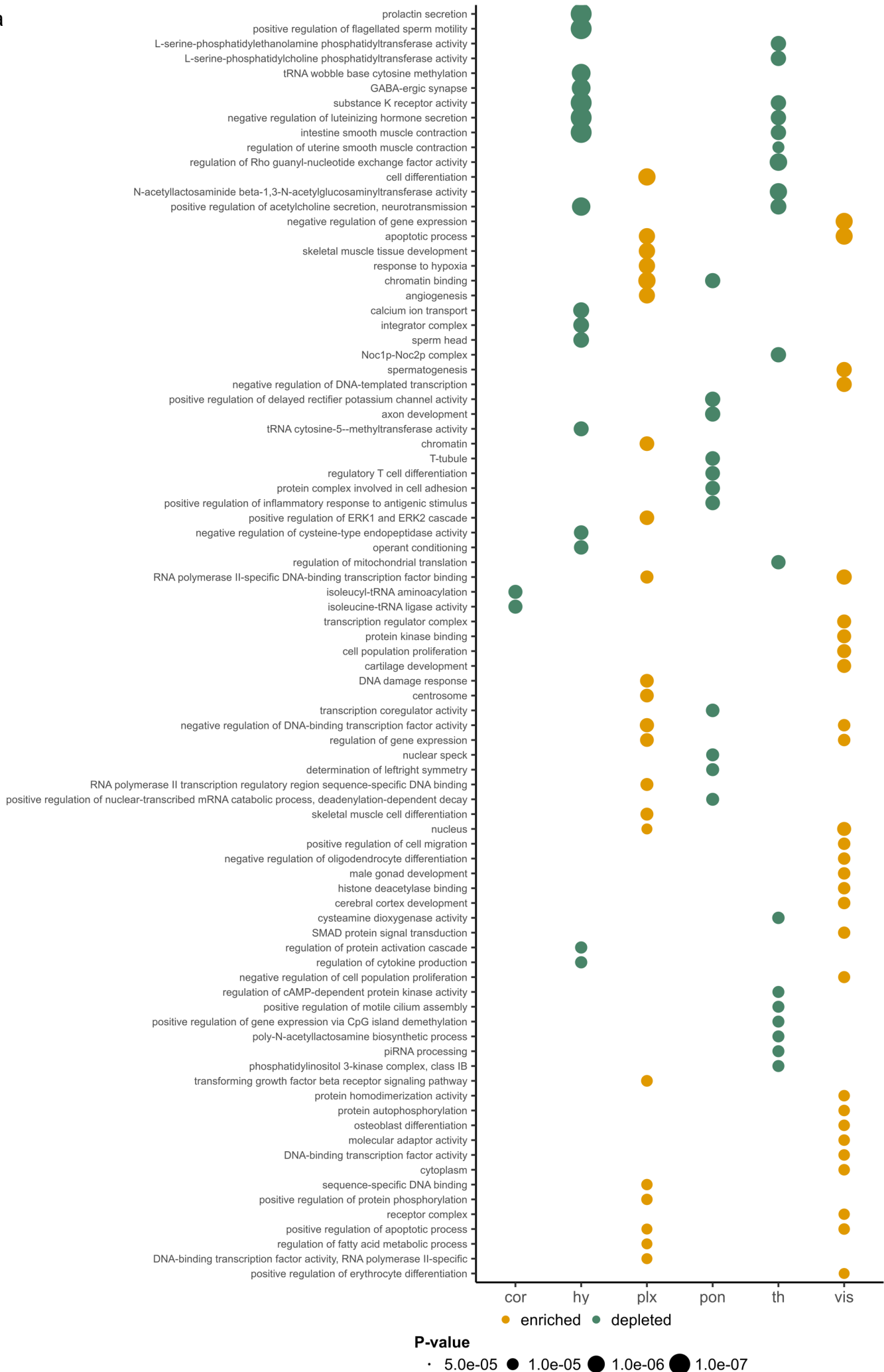

7a

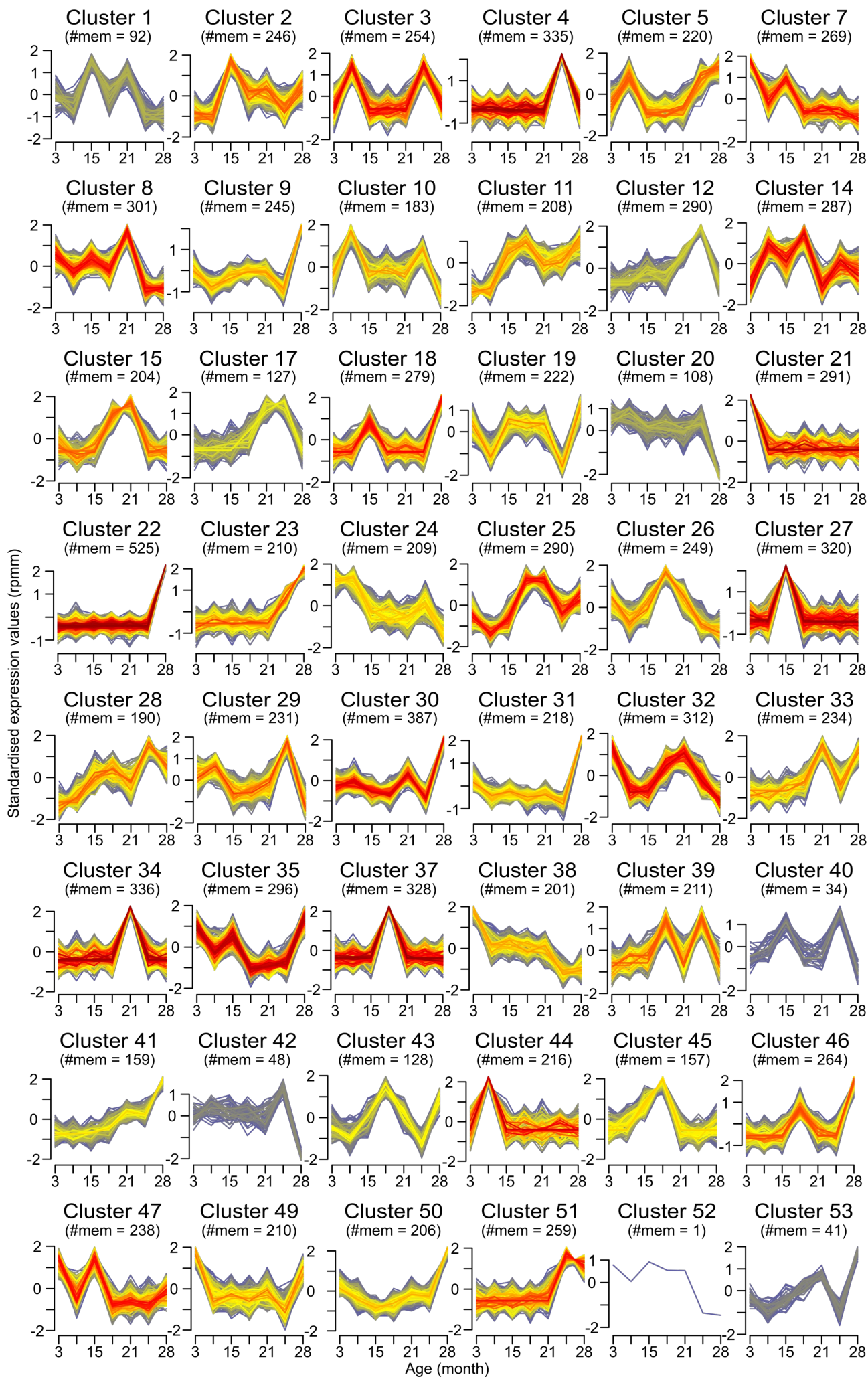

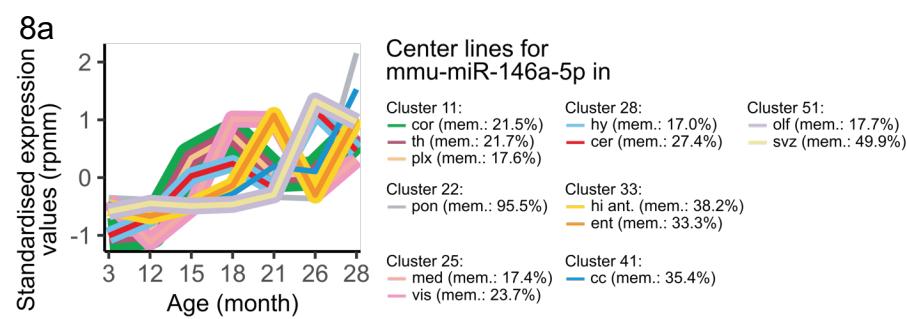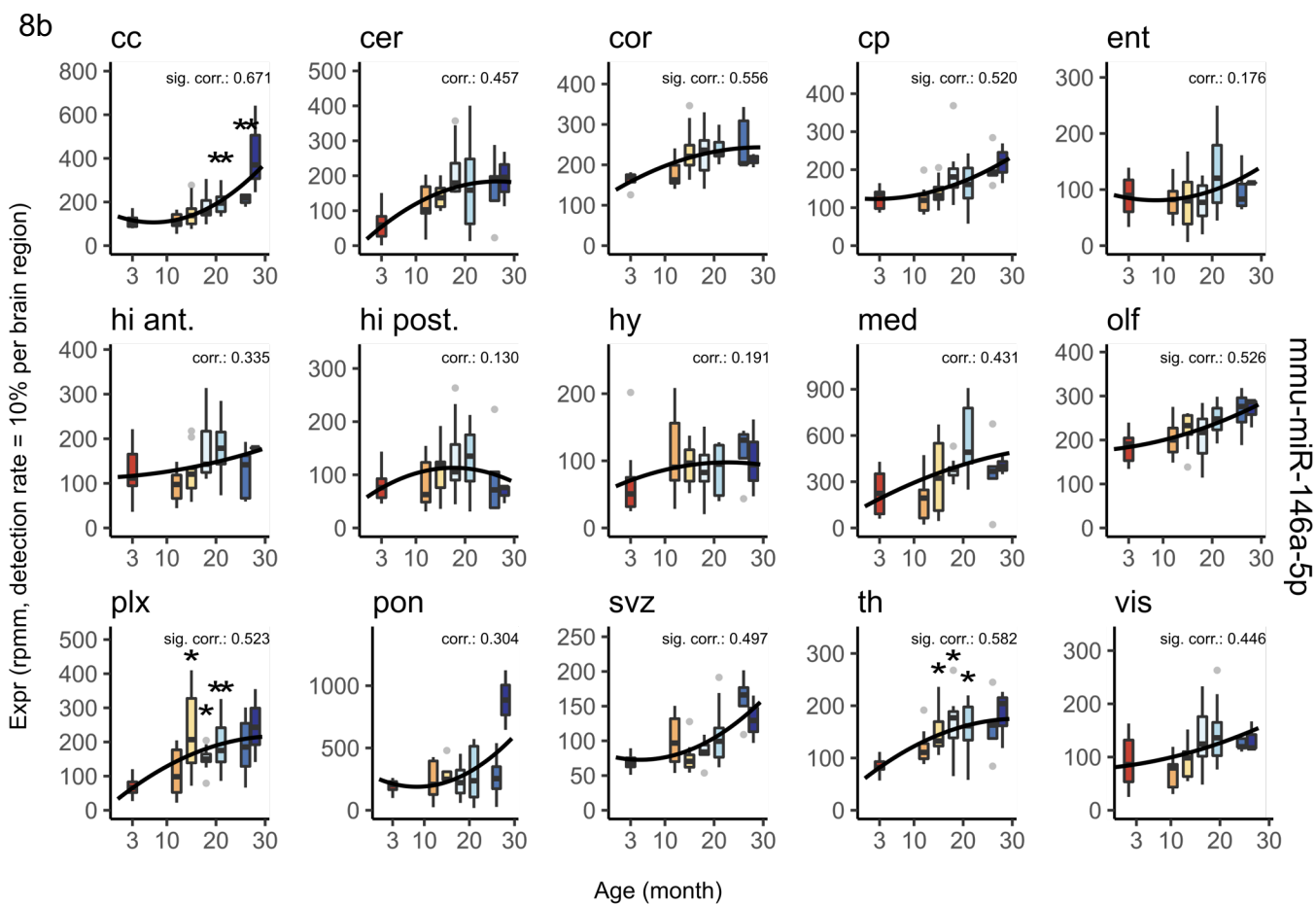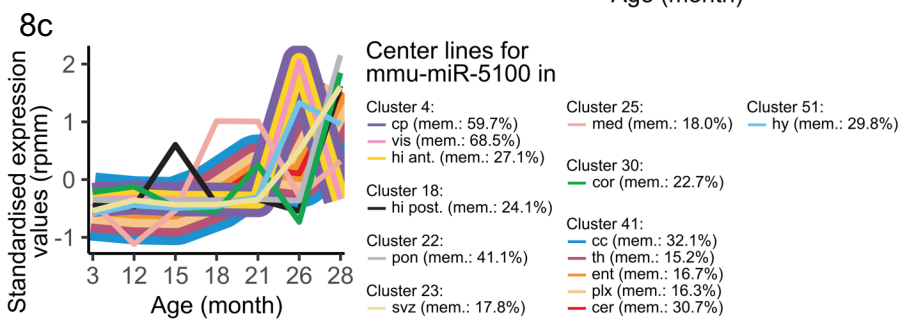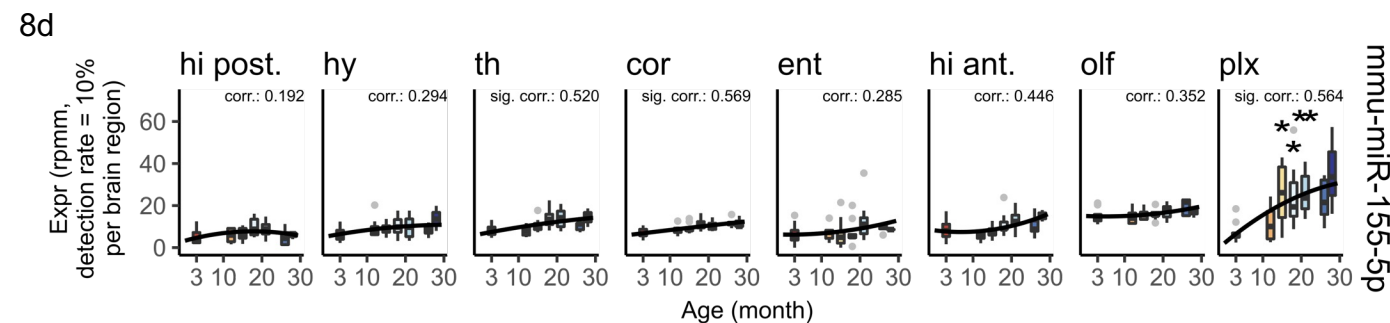

**9a**  
miR-146a-5p  
Brainstem versus:

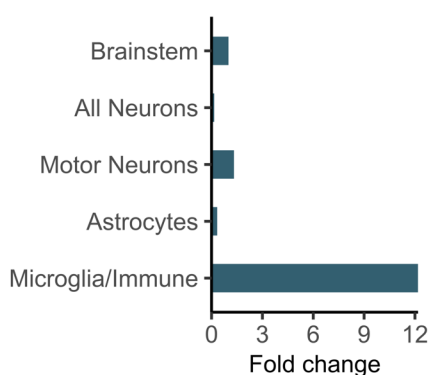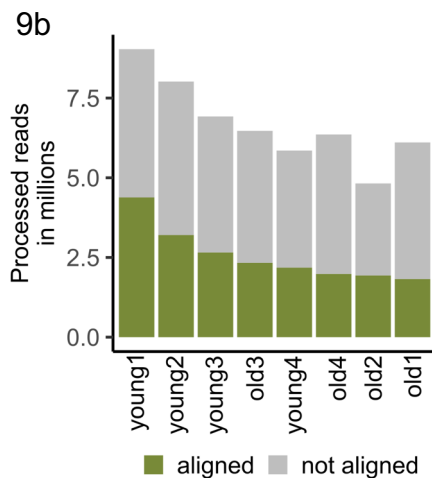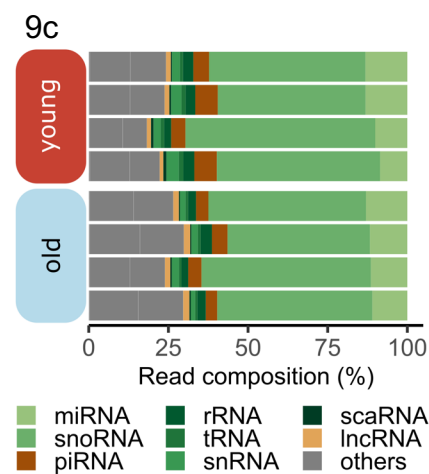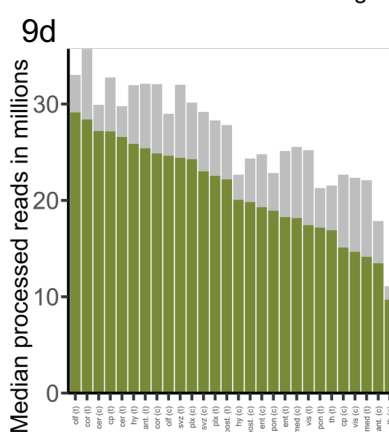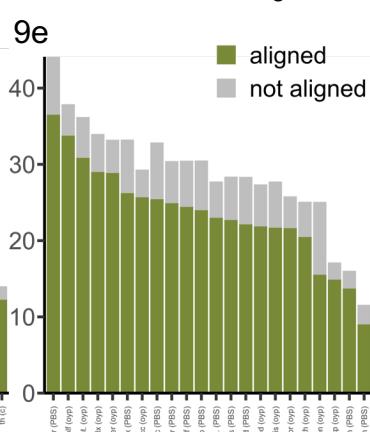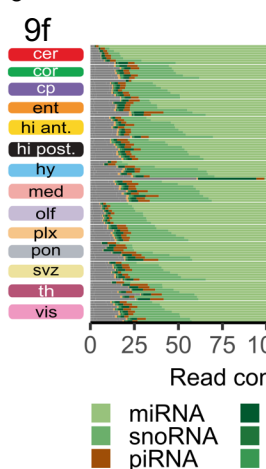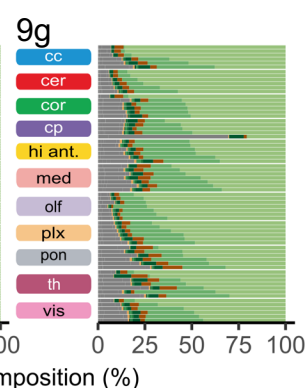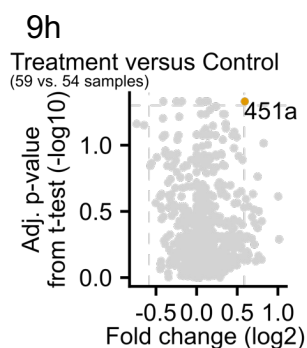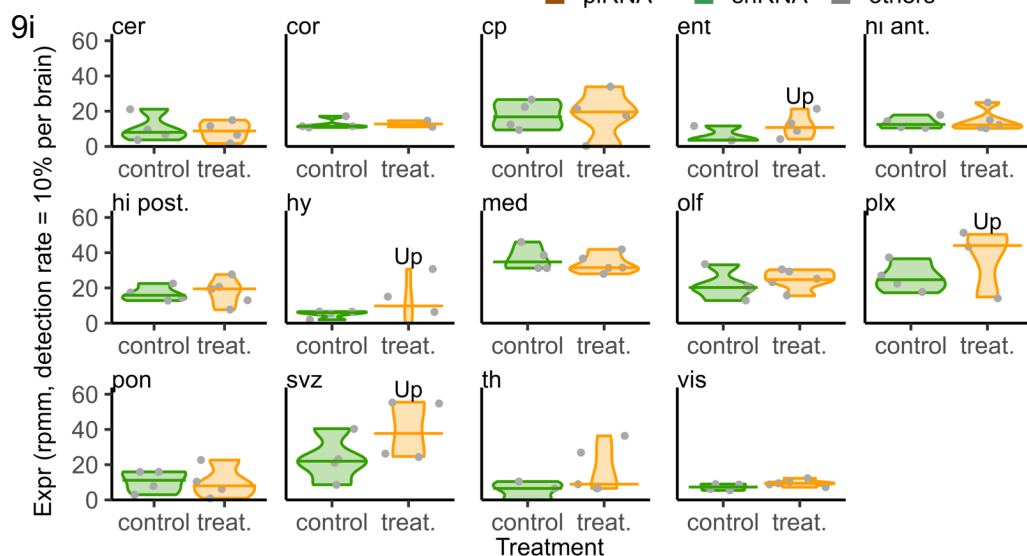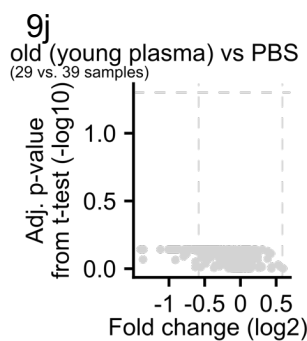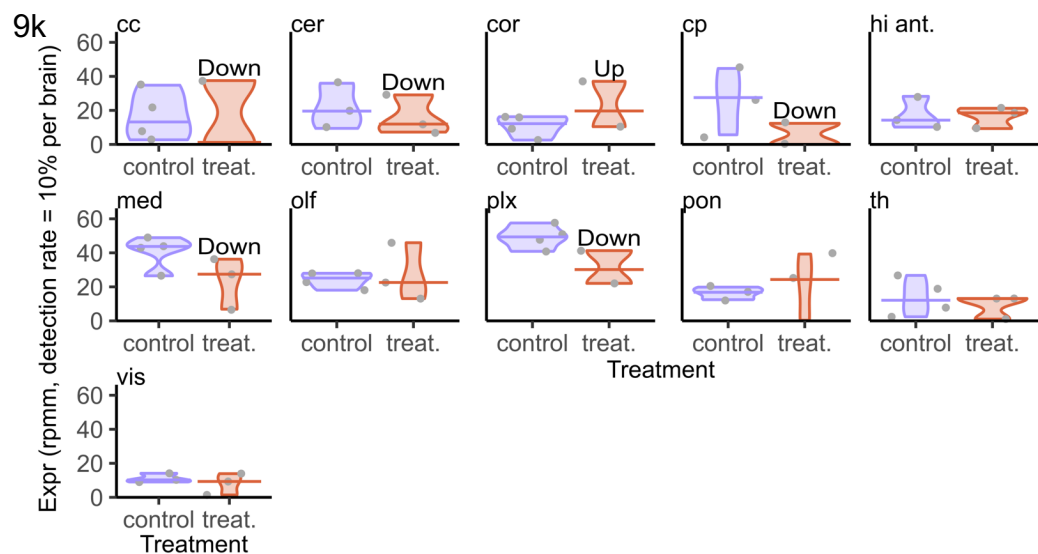

mmu-miR-155-5p

mmu-miR-155-5p

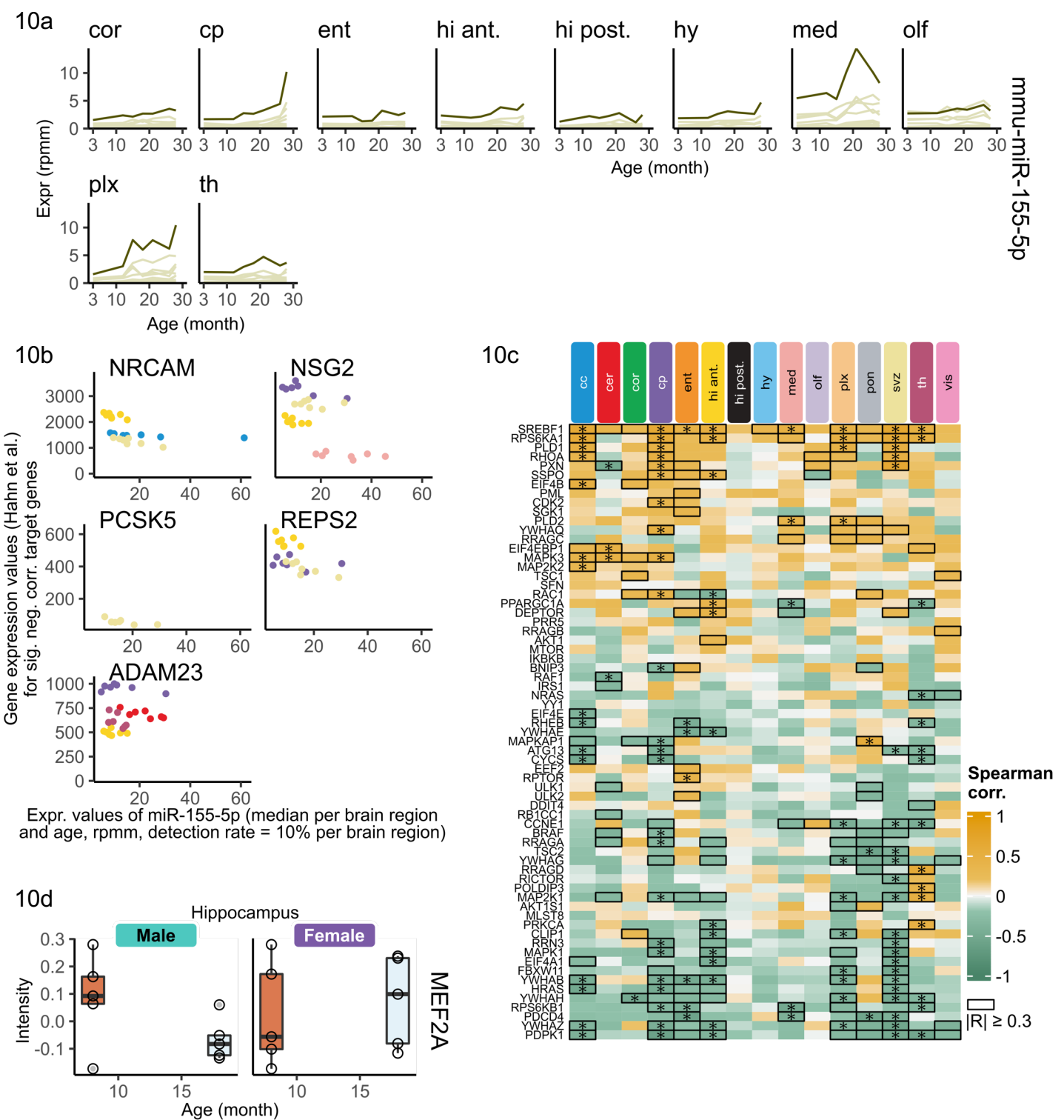

Supplement: Supplement 1 — Supplementary Fig. 1: a Overview of the aligned (green) and not aligned (grey) reads against the mouse genome. Combined per brain region and age. Values are given in millions. b Reads mapped to RNA types per sample. Brain regions are highlighted at the left side. c UMAP for all regions and all features (miRNAs, lncRNAs, piRNAs, rRNAs, scaRNAs, snoRNAs, snRNAs and tRNAs) colored by brain regions as indicated in Fig. 1a. d UMAP of all samples and all features colored by sex. Analogue to Supplementary Fig. 1c. e UMAP of all samples and features colored by age. Analogue to Supplementary Fig. 1c. f For each brain region and RNA class, we determined the composition of expressed RNA counts, considering only RNAs with raw counts ≥ 5 in at least 10% of all samples from that brain region. g For all brain regions and different RNA classes, we analyzed the composition of expressed RNA counts at each age point individually. The trends for all fifteen brain regions are visualized and lines are fitted with a third-degree polynomial. Supplementary Fig. 2: a UMAP of all samples and for all tRNAs colored by sex. Analogue to Fig. 1d. b UMAP of all samples and for all tRNAs colored by age. Analogue to Fig. 1d. c UMAP of all samples and for all miRNAs colored by sex. Analogue to Fig. 1f. d UMAP of all samples and for all miRNAs colored by age. Analogue to Fig. 1f. e Expressed miRNAs per brain region and the overlap between them. f UMAP of young samples (3-, 12-, and 15-month-old) colored by brain region. g UMAP of all young samples according to Supplementary Fig. 2f colored by sex. h UMAP of the young samples according to Supplementary Fig. 2f colored by age. i Analogue to Fig. 2a for male and female samples combined: Heatmaps of the 50 top miRNA from all brain regions determined by coefficient of variation calculated using the medians of the expression values of each brain region. Shown are the absolute standardized expression values (z-scores). The black borders are indicating the bin [file media-1.pdf]
